# Prevalence and clinical consequences of atelectasis in SARS-CoV-2 pneumonia: a computed tomography retrospective cohort study

**DOI:** 10.1186/s12890-021-01638-9

**Published:** 2021-08-17

**Authors:** Álvaro Mingote, Andrea Albajar, Paulino García Benedito, Jessica Garcia-Suarez, Paolo Pelosi, Lorenzo Ball, Javier García-Fernández

**Affiliations:** 1 Anaesthesia, Critical Care Department and Pain Unit, Puerta de Hierro Universitary Hospital - Majadahonda, c/Manuel de Falla, 1, 28222 Madrid, Spain; 2 Radiodiagnostic Unit, Puerta de Hierro Universitary Hospital – Majadahonda, Madrid, Spain; 3 Department of Surgical Sciences and Integrated Diagnostics, University of Genoa, Genoa, Italy; 4 Anesthesia and Critical Care, IRCCS for Oncology and Neurosciences, San Martino Policlinico Hospital, Genoa, Italy; 5 Autonomous University of Madrid, Madrid, Spain

**Keywords:** Atelectasis, Coronavirus, Chest computed tomography, Mechanical ventilation, Severe acute respiratory syndrome

## Abstract

**Background:**

The aim of the study is to estimate the prevalence of atelectasis assessed with computer tomography (CT) in SARS-CoV-2 pneumonia and the relationship between the amount of atelectasis with oxygenation impairment, Intensive Care Unit admission rate and the length of in-hospital stay.

**Patients and methods:**

Two-hundred thirty-seven patients admitted to the hospital with SARS-CoV-2 pneumonia diagnosed by clinical, radiology and molecular tests in the nasopharyngeal swab who underwent a chest computed tomography because of a respiratory worsening from Apr 1 to Apr 30, 2020 were included in the study. Patients were divided into three groups depending on the presence and amount of atelectasis at the computed tomography: no atelectasis, small atelectasis (< 5% of the estimated lung volume) or large atelectasis (> 5% of the estimated lung volume). In all patients, clinical severity, oxygen-therapy need, Intensive Care Unit admission rate, the length of in-hospital stay and in-hospital mortality data were collected.

**Results:**

Thirty patients (19%) showed small atelectasis while eight patients (5%) showed large atelectasis. One hundred and seventeen patients (76%) did not show atelectasis. Patients with large atelectasis compared to patients with small atelectasis had lower SatO_2_/FiO_2_ (182 vs 411 respectively, *p* = 0.01), needed more days of oxygen therapy (20 vs 5 days respectively, *p* = 0,02), more frequently Intensive Care Unit admission (75% vs 7% respectively, *p* < 0.01) and a longer period of hospitalization (40 vs 14 days respectively *p* < 0.01).

**Conclusion:**

In patients with SARS-CoV-2 pneumonia, atelectasis might appear in up to 24% of patients and the presence of larger amount of atelectasis is associated with worse oxygenation and clinical outcome.

**Supplementary Information:**

The online version contains supplementary material available at 10.1186/s12890-021-01638-9.

## Background

The use of chest computed tomography (CT) has shown a greater diagnostic sensitivity and specificity compared to chest-X ray in patients with SARS-CoV-2 pneumonia [1]. Three main phenotypes on chest CT have been described with potential implications for clinical management: multiple bilateral ground glass opacities (phenotype one), unhomogeneously distributed atelectasis with peribronchial infiltrates (phenotype two) and development of an ARDS-like pattern (phenotype three) [2]. Some studies [3] suggest that in the phenotype one, the compliance of the respiratory system is higher despite the patient's hypoxemia, thus lower to moderate levels of PEEP may redistribute pulmonary flow and reduce shunt. In the phenotype two and three, a progressive predominance of atelectasis occurs, which might benefit to moderate to higher levels of PEEP as well as prone position to recruit non-ventilated lung regions although other studies reported conflicting results [4, 5]. On the other hand, some of these studies have denied the existence of atelectasis in the SARS-CoV-2 pneumonia patient, expressing that they are exceptional (less than 5%), and clinically postulated that recruitment maneuvers are contraindicated [6]. We hypothesized that patients with SARS-CoV-2 pneumonia had higher prevalence of atelectasis and that larger compared to smaller amount of atelectasis were associated with worse oxygenation and poor clinical outcome.

The present study aims to estimate the prevalence of atelectasis in patients with SARS-CoV-2 pneumonia and determine whether the amount of these atelectasis may be associated with the clinical outcome in terms of oxygen therapy need, Intensive Care Unit admission rate, length of in-hospital stay and secondly, in-hospital mortality.

## Patients and methods

### Study population

**Fig. 1:**
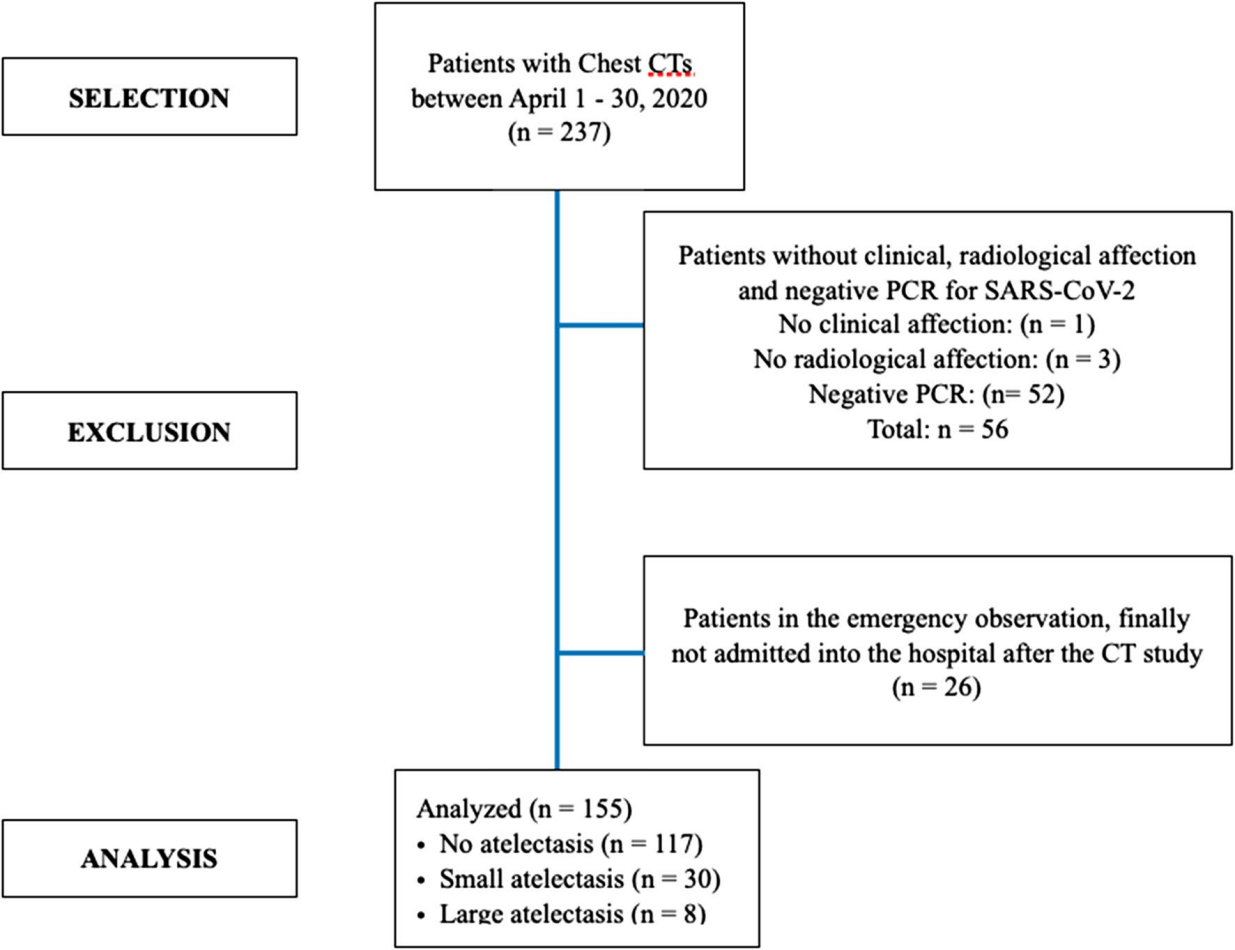
Flowchart of the patients included in the study

This retrospective study was approved by the Ethical Committee for Medical Research of Puerta de Hierro Majadahonda Universitary Hospital (Madrid, Spain) on 29 June 2020. The electronic medical records were reviewed and analyzed. All methods have been carried in accordance with current regulations and guidelines. All patients were aged > 18 years and informed consent was obtained before performing the chest CT. We analyzed 237 patients who underwent a contrast-enhanced chest-CT pulmonary angiography (CTPA) between April 1 and April 30, 2020. From this group of patients, we selected those patients who were admitted to the hospital with SARS-CoV-2 pneumonia diagnosed by clinical, radiology and molecular tests in the nasopharyngeal swab who underwent a chest computed tomography (Fig. 1). CTPA examinations were performed in multi-detector CT scanners (Aquilion Prime SP, Canon Medical Systems and Somatom Sensation, Siemens Healthineers) using a standard CTPA protocol. The whole chest was scanned from lowest hemidiaphragm to lung apex, in the supine position. All patients were instructed to hold breath, and CTPA images were acquired during a single breath-hold. Scan parameters were as follows: tube voltage of 120 kV, tube current of 100–300 mA·s, collimation of 64 × 0.6–0.625 mm, pitch of 0.937–1.0, gantry rotation time of 0.5 s. Nonionic iodinated contrast media, (50–70 mL, iomeprol 400 mg/mL) was injected via an antecubital vein at a flow rate of 4 mL/s followed by a 25 mL saline flush using a mechanical power injector. Automatic bolus-tracking technique with the region of interest positioned at the level of the main pulmonary artery and a trigger threshold of 120 HU, and a fixed delay of 5 s was employed. Images were reconstructed at 3 mm thickness in axial and coronal planes. Source images were transmitted to workstations for multiplanar reconstructions and to picture archiving and communication systems (PACS).

**Fig. 2:**
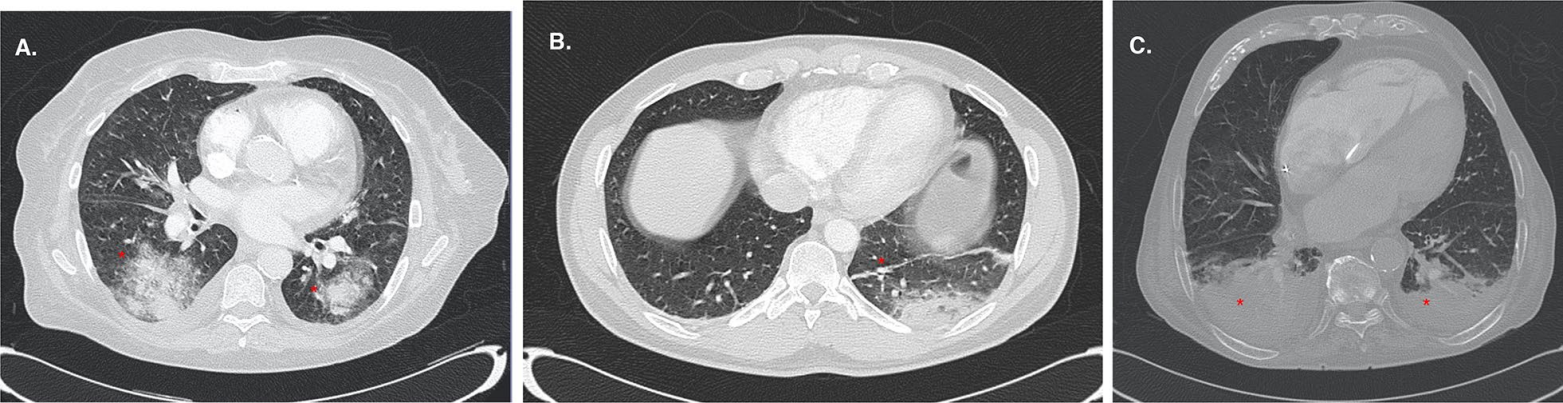
Representative chest Computed Tomography scans in patients with SARS-CoV-2 pneumonia. **a** Patient without atelectasis: multiple ground-glass opacities in both lungs (red asterisks), as well as a minimum pleural effusion that do not condition atelectasis. **b** Patient with small atelectasis. Well-defined, linear opacity is seen in the left lower lobe (red asterisk), corresponding to laminar atelectasis (middle panel). **c** Patient with large atelectasis. A triangular opacity in both inferior lobes (red asterisk) corresponding to atelectasis is observed, surrounded by pleural effusion in the same location (right panel)

After the analysis of the CT study, the patients were classified into three groups (Figs. 1, 2): (a) those who did not present atelectasis, (b) those who presented *small atelectasi*s (laminar, subsegmentary or segmentary atelectasis with estimated size of less than 5% of the forecasted lung volume) (*small atelectasis* hereinafter) and (c) those with significant atelectasis (atelectasis with estimated size of more than 5% of the forecasted lung volume) or a complete lobe collapse (*large atelectasis* hereinafter). In this sense, one radiologist specialized in SARS-CoV-2 pneumonia performed a visual assessment based on the single slice in the CT with the largest atelectasis size and compared it to the estimated lung volume, classifying the patients into these three groups. We choose the 5% of lung surface as a cut-off point according to our capacity to detect and quantitate the % of lung with atelectasis. Table 1 shows basal characteristics of the patients that accomplished the inclusion criteria (including time from the beginning of symptoms).

**Table 1:** Basal characteristics of the patients that accomplished the inclusion criteria

	No atelectasis	Small atelectasis	Large atelectasis	Overall *p*-value	No atelectasis versus small atelectasis *p*-value	No atelectasis vs large atelectasis *p*-value
N (%)	117 (75)	30 (19)	8 (6)	–	–	–
*Sex (%)*				0.36	0.37	0.68
Male	85 (73)	19 (63)	7 (88)			
Women	32 (27)	11 (37)	1 (12)			
Age (years)	68.7 ± 13.9	68.4 ± 15.7	70 ± 14.2	0.98	0.95	0.83
BMI > 30 kg/m^2^ (%)	23 (21)	6 (20)	0 (0)	0.38	0.99	0.35
Women	11 (48)	3 (50)	–			
Men	12 (52)	3 (50)	–			
Days with symptoms until CT (days)	18.0 ± 9.6	15.8 ± 10.8	17.6 ± 6.9	0.55	0.28	0.92
CURB-65 scale	1 (1 to 2)	1 (0 to 2)	2 (2 to 2)	0.1	0.12	0.35
Chest CT severity scale	14 (8 to 17)	11 (7 to 15)	18 (12 to 21)	0.02*	0.01*	0.29
*Blood parameters*						
D Dimer (ug/mL)	2.7 (1.4 to 2.1)	1.9 (0.8 to 3.3)	3.8 (2.7 to 7.4)	0.03*	0.05	0.15
Fibrinogen (mg/dL)	490 (321 to 624)	452 (383 to 581)	418 (324 to 478)	0.47	0.85	0.22
Neutrophils (10^9^/l)	7.43 (4.37 to 9.98)	5.94 (3.41 to 9.90)	10.0 (6.05 to 13.0)	0.15	0.21	0.17
IL—6 (pg/mL)	24.6 (2.7 to 97.1)	13.1 (4.5 to 30.7)	20.6 (15.3 to 1497.0)	0.35	0.24	0.51
SatO_2_/FiO_2_	357 (230 to 438)	411 (331 to 453)	182 (113 to 359)	0.01*	0.07	0.17
*Type of ventilatory support (%)*						
No ventilatory support	17 (14)	9 (30)	0 (0)	–	–	–
Nasal cannula	44 (38)	11 (37)	1 (12)			
Reservoir mask	33 (28)	6 (20)	1 (12)			
High flow nasal cannula	5 (4)	1 (3)	0 (0)			
Non-invasive ventilation	1 (1)	1 (3)	0 (0)			
Invasive mechanical ventilation	17 (15)	2 (7)	6 (75)			
In-hospital mortality (n. %)	13 (11)	2 (7)	1 (12)	0.76	0.74	0.99

*p*-value indicates if differences between the three groups were found or not. Age and days are mean and standard deviation. Clinical (CURB 65 scale), radiological (Chest Computed Tomography scale) and blood parameters are measured at the time of chest Computed Tomography, and show median, p25 and p75 quartiles. BMI: Body Mass Index; CT: Computed Tomography; IL-6: interleukin – 6; SatO_2_/FiO_2_: ratio between peripheral oxygen saturation and inspiratory oxygen fraction

* *p*-value < 0.05

### Clinical and radiological severity

In order to assess the clinical severity of pneumonia, the patient's score on the CURB 65 scale was collected upon hospital admission [7] [see Additional file 1]. One radiologist (the same for all patients) specialized in SARS-CoV-2 pneumonia thoracic imaging studies used a semi-quantitative scoring system with a visual assesment [8] to estimate the pulmonary involvement of all these abnormalities on the basis of the area involved, based on the chest CT findings described by Wong et al. in 2003 and 2004 [9, 10]. Each of the five lung lobes was visually scored on a scale of 0 to 5: 0: no involvement; 1: less than 5% involvement; 2: 5–25% involvement; 3: 26–49% involvement; 4: 50–75% involvement; 5: more than 75% involvement. The total CT score was the sum of the individual lobar scores and ranged from 0 (no involvement) to 25 (maximum involvement). The patients underwent the chest CT study because of a respiratory worsening (decrease Pa/Fi O_2_, increase in oxygen therapy support) and in no circumstance for scientific purposes.

### Oxygenation and oxygen therapy need

Sat O_2_/FiO_2_ ratio from each patient was collected as an indirect estimate of lung oxygenation capacity [11] at two moments: at the time of the chest CT and the worse ratio during the hospitalization. The number of days that each patient needed to maintain the most intensive oxygen therapy (the one with the greatest flow and FiO_2_) received during admission was also collected.

### Other variables with prognostic interest

In addition, outcome variables that have value when it comes to the analysis of the prognosis, such as in-hospital mortality, days of admission to the ICU (if required) or total days of hospitalization were collected. We adjusted in-hospital mortality based on the presence of pulmonary embolism (PE) by comparing the mortality between the groups with PE versus no PE (since the reason for inclusion in the study was the performance of a chest CT due to a respiratory worsening, including suspect of PE associated with SARS-CoV-2 pneumonia, Additional file 1).

### Data storage

After obtaining the consent of the ethics committee to record the data from the informatic patient’s clinical reports, a database using the software FileMaker Pro version 18.0.3.317 in a MacOS operating system was organized. In this database, the personal data of the patients were adequately encoded to guarantee data protection.

### Statistical processing

Data from the prevalence of atelectasis, ICU admission rate and mortality are shown as percentage of the group. Lung oxygenation capacity, length of in-hospital stay and days with higher oxygen therapy are shown as median (p25, p75). For the analysis of quantitative variables, a normality test was carried out and then an ANOVA test was performed to test if there were differences between the three groups (or a Kruskal Wallis test if the data set did not fit the normal distribution). If this analysis was significant, two-by-two post hoc comparisons (*Bonferroni test or Dunn’s test*) were carried out between the groups in order to correct for multiple comparisons. For the analysis of qualitative variables, after checking if the collected variable met the minimum characteristics required, a χ^2^ test was done. If this analysis was significant, two-by-two post hoc comparisons (χ^2^ or Fisher test) were carried out between the groups. Statistical analysis was carried out using Prism Graphpad version 8.0 software. Statistical significance was assumed for two-tailed *p* < 0.05. Sensitive data of the patients (name, surname, medical record number, etc.) transferred to this software, were duly encoded.

## Results

From the 237 patients analyzed, 155 patients (111 males and 44 females; mean age 68.8 ± 13.9 years) met the inclusion criteria. Basal characteristics of the patients are shown in Table 1. Indeed, up to 38 patients (24%) showed some degree of atelectasis at chest CT: 30 (19%) patients showed small atelectasis in the chest CT and 8 patients (5%) showed large atelectasis.

**Fig. 3:**
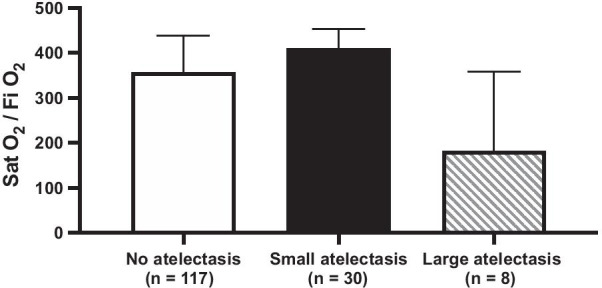
SatO_2_/FiO_2_ ratio at the time of CT. Columns show the median score (357, 411 and 182 respectively) and bars show the p75, *n* shows number of patients from each group

SatO_2_/FiO_2_ ratio differences were found among the groups (*p* < 0.01) (Fig. 3). In the group without atelectasis, we found a SatO_2_/FiO_2_ ratio of 357 (230 to 438), compared to 411 (331 to 453) in those with small atelectasis (*p* = 0.07) and to 182 (113 to 359) in those with large atelectasis (*p* = 0.17). SatO_2_/FiO_2_ ratio was lower in patients with large compared to small atelectasis (*p* = 0.01).

The worst SatO_2_/FiO_2_ ratio was different among groups (*p* = 0.03), being 300 (range 99 to 431) in the patients without atelectasis, 379 (range 247 to 453) in patients with small atelectasis (*p* = 0.05), and 182 (range 113 to 359) in patients with large atelectasis (*p* = 0.37). The worst SatO_2_/FiO_2_ ratio was lower in patients with large compared to small atelectasis (*p* = 0.05).

In addition, in the no atelectasis group and in the small atelectasis group, the need for Intensive Care Unit admission rate was lower compared to large atelectasis group (*p* < 0.01). In this sense, in the group of patients without atelectasis 18 patients (15%) needed to be admitted in the ICU, compared to 2 patients (7%) in the group of small atelectasis (*p* = 0.37) and to 6 patients (75%) in the group of larger atelectasis (*p* < 0.01). Indeed, ICU admission rate was lower in patients with small atelectasis compared to large atelectasis (*p* < 0.01).

**Fig. 4:**
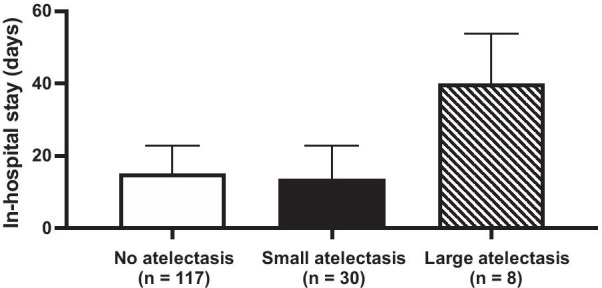
Length of in-hospital stay (days). Columns show the median score (15, 14 and 40 days respectively) and bars show the p75, *n* shows number of patients from each group

The length of in-hospital stay was different among groups (*p* = 0.01). Patients with no atelectasis required 15 (7 to 23) days of in-hospital admission, compared to 14 (6 to 23) days in patients with small atelectasis (*p* = 0.99) and to 40 (17 to 54) days in patients with large atelectasis (*p* = 0.02) (Fig. 4). The length of in-hospital stay was longer in patients with large compared to small atelectasis (*p* = 0.01).

The duration of oxygen therapy was different among groups (*p* = 0.04). Patients without atelectasis needed to maintain it for 6 (3 to 10) days, compared to 5 (5 to 10) days in patients with small atelectasis (*p* = 0.48), and to 20 (4 to 37) days in those with large atelectasis (*p* = 0.04). Furthermore, differences were found between these last two groups (*p* = 0.02).

In-hospital mortality was not different among groups (*p* = 0.55). The percentage of patients who died in-hospital was 11% in patients without atelectasis compared to 6,7% in patients with small atelectasis and 12.5% of patients with large atelectasis. Adjusting for PE, (see Additional file 1 for more information), in the no atelectasis group, 19% of the patients with PE deceased compared to 6.9% of mortality in those who did not have PE. In patients with small atelectasis, no patients with PE deceased (0%), while 8.3% of the patients died in the group without PE. In the group of patients with large atelectasis 33% of patients with PE died while none (0%) of patients without PE died in-hospital.

## Discussion

In the present study we found that in patients with SARS-CoV-2 pneumonia the prevalence of atelectasis was 24%. Among them, 19% of the patients showed small atelectasis and 5% of the patients showed large atelectasis. Patients with larger compared to smaller atelectasis showed less SatO_2_/FiO_2_ ratio, higher rate of ICU admission and longer length of in-hospital stay. Among the few published studies investigating atelectasis in chest CT in SARS-CoV-2 pneumonia patients, our study includes the largest number of patients (237 patients screened, of whom 155 patients met all entry criteria). In our study, the prevalence of atelectasis was 24% which is higher than the previously reported (around 5%) [12]. However, previous studies did not focus on atelectasis but generally describing the most frequently patterns at chest CT scans [13, 14]. Furthermore, the greatest prevalence of atelectasis was mostly composed by mild, segmental or subsegmental atelectasis. No previous study separated smaller and larger atelectasis associated with the clinical outcome. We made this distinction based on proposed pathophysiology of the SARS-CoV-2 pneumonia and its relationship with oxygenation: it has been suggested that the progression of the disease would be associated with more severe hypoxia [15]. Currently and from anatomopathological studies carried out on patients with ARDS, we know that in addition to diffuse alveolar injury and hyaline membrane formation (typical of all ARDS and VILI), there are areas of dead space that worsen gas exchange even more [16]. Moreover, the hypoxic pulmonary vasoconstriction reflex (Euler-Liljestrand mechanism [17]) may further worsen ischemic and thrombotic phenomena in the lung. Patients with large atelectasis showed a tendency to a greater involvement of lung tissue by SARS-CoV-2 pneumonia than in those without and small atelectasis. In fact, they required a higher ICU admission rate, with greater need of intubation and invasive mechanical ventilation. In addition, patients with larger atelectasis showed worse oxygenation ratios (Sat O_2_/FiO_2_) during admission and had a longer in-hospital stay (nearly the double than in patients with small or no atelectasis). Previous studies reported a lower incidence of atelectasis in patients with COVID-19 pneumonia [3]. Our data suggest that the presence of large atelectasis may be a factor affecting progressive evolution to the ARDS [16]. The Sat O_2_/FiO_2_ ratio and atelectasis detection by chest CT might be two valuable tools for the lung function assessment of these patients and hence might be helpful to predict those patients who will need mechanical ventilation, ICU admission and prolonged length of in-hospital stay. In-hospital mortality was not different among patients with no, small or large atelectasis. This can be explained by the fact that mortality rate of SARS-CoV-2 pneumonia is not only related to pulmonary injury, but also to development of multiple organ failures [18]. The development of significant atelectasis favors more chances of respiratory complications and a higher morbidity with greater risk of ICU admission and need for invasive mechanical ventilation. The establishment of an early treatment with non-invasive respiratory ventilation to reverse atelectasis or prevent their progression, together with intensive surveillance or in intermediate care, could prevent a progression of lung injury with a worse outcome in patients in which this condition is detected.

Our study has several limitations to be addressed. First, this is a retrospective study, but in a situation of healthcare collapse, it was impossible to carry out prospective studies which need more time for approval and organization. However, our hospital has all the computerized medical history data, making data collection very reliable. We do recognize that a number of patients that were included in the study did not have a specific medical record (it was even, in a number of cases, the first time to come to the hospital). Second, we selected all the chest CT scans in the period with the highest incidence of SARS-CoV-2 pneumonia in our hospital, with high clinical suspicion of PE. Additionally, the scale we used to measure the severity of the pulmonary affection in chest CT ([8], shown in Additional file 1), based on the findings of Wong KT et al. [9, 10], takes into account the percentage of lobar infiltration and even pleural effusion, which could be logically associated with worst outcome. However, there were no differences between the large atelectasis group and the other groups in this scale. Regarding atelectasis, the same radiologist carried out a visual quantification by lobes for every scan in order to reduce heterogeneity between various radiologists. Third, the group without atelectasis might not be considered as a standard control group but a group of patients with significant oxygenation impairment due to several causes (including PE), hence introducing a possible selection bias as patients without atelectasis and mild infection would not be represented. However, this makes our study's findings even more relevant in relation to the differences between the group of large atelectasis (which indeed is composed by a reduced number of patients, 8) and its relationships with outcome compared with the other groups. We did not evaluate complications occurring during the hospital stay, but we do know that there are several differences between the groups regarding oxygenation, oxygen-therapy needs, ICU admission and days of hospitalization. Whether if these differences are associated directly or indirectly to atelectasis has to be determined on further studies.

## Conclusions

In patients with SARS-CoV-2 pneumonia, atelectasis might appear in up to 24% of patients and the presence of larger amount of atelectasis is associated with worse oxygenation and clinical outcome. Patients with larger atelectasis requires more intensive surveillance and might benefit of open lung techniques.

## Supplementary Information

**Additional file 1**. Scores used in the study & Analysis of in-hospital mortality adjusted to pulmonary embolism (PE).

## Data Availability

The datasets used and/or analyzed during the current study are available from the corresponding author on reasonable request.

## Industry relationships for past 2 years

The authors declare no specific relationships.
