# Supplementary material for: Prevalence and clinical consequences of atelectasis in SARS-CoV-2 pneumonia: a computed tomography retrospective cohort study
Source: BMC Pulm Med. 2021 Aug 17;21:267. doi: 10.1186/s12890-021-01638-9 (PMC8369136; doi:10.1186/s12890-021-01638-9)
Supplement: Supplementary file 1 — Additional file 1. Scores used in the study & Analysis of in-hospital mortality adjusted to pulmonary embolism (PE). [file 12890_2021_1638_MOESM1_ESM.docx]

**Prevalence and clinical consequences of atelectasis in SARS-CoV-2 pneumonia:**

**a computed tomography retrospective cohort study.**

*Mingote Lladó, A.^1^; Albajar, A. ^1^; García Benedito, P.^2^; García-Suárez, J.^1^; Pelosi, P^,3,4^ ; Ball, L^3,4^; García-Fernández, J.* ^1,5^

^1^ Anaesthesia, Critical Care Department and Pain Unit, Puerta de Hierro Universitary Hospital -Majadahonda, Madrid - Spain.

^2^ Radiodiagnostic Unit, Puerta de Hierro Universitary Hospital – Majadahonda, Madrid - Spain.

^3^ Department of Surgical Sciences and Integrated Diagnostics, University of Genoa, Genoa, Italy

^4^ Anesthesia and Critical Care, San Martino Policlinico Hospital, IRCCS for Oncology and Neurosciences, Genoa, Italy

^5^ Autonomous University of Madrid, Madrid, Spain.

Correspondence: Álvaro Mingote Lladó, Anaesthesia, Critical Care Department and Pain Unit in Puerta de Hierro Universitary Hospital – Majadahonda . c/Manuel de Falla, 1. 28222 Madrid, Spain. e-Mail: [alvaro.mingote.llado@gmail.com](mailto:alvaro.mingote.llado@gmail.com)

SUPPLEMENTARY MATERIAL

**APPENDIX**

**Appendix I: Scores used in the present study**

- **CURB65 (from Barlow et al.):**

Based on the presence or absence of the following criteria (1 point per each):

New confusion

Urea .7 mmol/l

Respiratory rate >30/min

Systolic blood pressure < 90 mm Hg or diastolic blood pressure < 60 mm Hg

Age >65 years

Depending on the score, the *patient may be classified as*:

Severe = 3 or more points.

Non-severe (moderate risk) = 2 points.

Non-severe (low risk) = 0 or 1 point.

- **Radiological severity COVID19 scale (based on Pan F. et al):**

A semi-quantitative scoring system by Pan F et al. was used to estimate the pulmonary involvement of all these abnormalities on the basis of the area involved, based on the chest CT findings described by Wong KT et al in 2003 and 2004. Each of the five lung lobes was visually scored on a scale of 0 to 5:

0: no involvement

1: less than 5% involvement

2: 5%–25% involvement

3: 26%–49% involvement

4: 50%– 75% involvement

5: more than 75% involvement.

The total CT score was the sum of the individual lobar scores and ranged from 0 (no involvement) to 25 (maximum involvement).

**Appendix II: In hospital mortality, atelectasis and pulmonary embolism (PE)**

**Figure II.1.** Percentage (%) of PE in the groups.

Columns show % of prevalence in each group,

*n* shows number of patients from each group.

**Figure II.2.** Percentage (%) of in-hospital mortality adjusted by PE.

Columns show % of in-hospital mortality in each group,

*n* shows number of patients from each group.
